# Long-term NMDAR antagonism correlates reduced astrocytic glutamate uptake with anxiety-like phenotype

**DOI:** 10.3389/fncel.2015.00219

**Published:** 2015-06-03

**Authors:** Eduardo R. Zimmer, Vitor R. Torrez, Eduardo Kalinine, Marina C. Augustin, Kamila C. Zenki, Roberto F. Almeida, Gisele Hansel, Alexandre P. Muller, Diogo O. Souza, Rodrigo Machado-Vieira, Luis V. Portela

**Affiliations:** ^1^Department of Biochemistry, Universidade Federal do Rio Grande do SulPorto Alegre, Brazil; ^2^Department of Physiology, Universidade Federal de SergipeSão Cristovão, Brazil; ^3^Laboratory of Exercise, Biochemistry and Physiology, Universidade do Extremo Sul CatarinenseCriciúma, Brazil; ^4^Laboratory of Neuroscience, LIM-27, Institute and Department of Psychiatry, Universidade de São PauloSão Paulo, Brazil; ^5^Center for Interdisciplinary Research on Applied Neurosciences (NAPNA), Universidade de São PauloSão Paulo, Brazil; ^6^Experimental Therapeutics and Pathophysiology Branch, National Institute of Mental Health, National Institutes of HealthBethesda, MD, USA

**Keywords:** anxiety, astrocytes, behavior, glutamate, memantine

## Abstract

The role of glutamate *N*-methyl-D-aspartate receptor (NMDAR) hypofunction has been extensively studied in schizophrenia; however, less is known about its role in anxiety disorders. Recently, it was demonstrated that astrocytic GLT-1 blockade leads to an anxiety-like phenotype. Although astrocytes are capable of modulating NMDAR activity through glutamate uptake transporters, the relationship between astrocytic glutamate uptake and the development of an anxiety phenotype remains poorly explored. Here, we aimed to investigative whether long-term antagonism of NMDAR impacts anxiety-related behaviors and astrocytic glutamate uptake. Memantine, an NMDAR antagonist, was administered daily for 24 days to healthy adult CF-1 mice by oral gavage at doses of 5, 10, or 20 mg/kg. The mice were submitted to a sequential battery of behavioral tests (open field, light–dark box and elevated plus-maze tests). We then evaluated glutamate uptake activity and the immunocontents of glutamate transporters in the frontoparietal cortex and hippocampus. Our results demonstrated that long-term administration of memantine induces anxiety-like behavior in mice in the light–dark box and elevated plus-maze paradigms. Additionally, the administration of memantine decreased glutamate uptake activity in both the frontoparietal cortex and hippocampus without altering the immunocontent of either GLT-1 or GLAST. Remarkably, the memantine-induced reduction in glutamate uptake was correlated with enhancement of an anxiety-like phenotype. In conclusion, long-term NMDAR antagonism with memantine induces anxiety-like behavior that is associated with reduced glutamate uptake activity but that is not dependent on GLT-1 or GLAST protein expression. Our study suggests that NMDAR and glutamate uptake hypofunction may contribute to the development of conditions that fall within the category of anxiety disorders.

## Introduction

Anxiety disorders are among the most prevalent psychiatric conditions worldwide. These disorders have been associated with social isolation, alcoholism, and increased suicide attempts and are also considered to be risk factors for the development of additional psychiatric disorders (Gross and Hen, 2004). Hence, it is imperative to understand the neurobiological mechanisms that are associated with anxiety disorders. It has recently been proposed that a functional imbalance of the tripartite glutamatergic synapse plays a role in anxiety disorders (Clement and Chapouthier, 1998; Nutt and Malizia, 2001; Nemeroff, 2003; Machado-Vieira et al., 2009, 2012). Indeed, glutamatergic neurotransmission offers multiple potential pharmacological targets for treating anxiety-related disorders, such as postsynaptic receptor signaling, presynaptic glutamate release, and astrocytic glutamate uptake (Szabo et al., 2009; Zarate et al., 2010; Riaza Bermudo-Soriano et al., 2012; Pilc et al., 2013).

Currently, antagonism of *N*-methyl-D-aspartate receptor (NMDAR) has been proposed as a feasible strategy for reducing the major symptoms that are linked to anxiety-like behavior (Cortese and Phan, 2005). Indeed, when memantine, an NMDAR antagonist, is administered to patients presenting with depression, anxiety or obsessive-compulsive disorder, their neuropsychiatric symptoms appear to be relieved (Tariot et al., 2004; Sani et al., 2012). By contrast, a recent work demonstrated that chronic antagonism of NMDAR induces elevated anxiety in healthy mice (Hanson et al., 2014). Overall, the current data that are available regarding the association between the use of NMDAR antagonists and the presentation of anxiety-related behaviors refute a simple model of dose-effect and instead seem to be closely related to the regimen, type of drug, or route of administration (Silvestre et al., 1997; Riaza Bermudo-Soriano et al., 2012; Schwartz et al., 2012). Additionally, it is prudent to consider that glutamatergic neurotransmission involves not only neuronal receptors (ionotropic and metabotropic) but also astroglial transporters that participate in neuron-astrocyte coupling.

Two major astroglial Na^+^-dependent glutamate transporters, glutamate transporter 1 (GLT-1, also known as EAAT2) and glutamate aspartate transporter (GLAST, also known as EAAT1), take up glutamate from synapses to maintain the homeostasis that is necessary to orchestrate the physiological activity of receptors (Danbolt, 2001). Remarkably, cerebral GLT-1 and GLAST are predominately localized in astrocytes, with very low expression in other cell types (Zhou and Danbolt, 2014). Moreover, astrocytes account for 95% of the glutamate uptake activity in the brain (Danbolt et al., 1992; Lehre and Danbolt, 1998). Importantly, a recent work demonstrated that cerebral microinjection of the GLT-1 inhibitor, dihydrokainic acid (DHK), induced anhedonia and anxiety in rats (John et al., 2015). Thus, one could claim that astrocytic dysfunction may have a considerable impact on the expression of anxiety-like phenotypes (Bechtholt-Gompf et al., 2010; Schroeter et al., 2010; Lee et al., 2013). Based on the principles of neuron-astrocyte coupling, we hypothesized that long-term antagonism of NMDAR would impact astrocytic function and that this would likely affect anxiety phenotype.

In this study, we aimed to investigate the impact of long-term NMDAR antagonism by memantine on anxiety-related paradigms and their potential association with astrocytic glutamate transport.

## Materials and Methods

### Animals

Three-month-old CF-1 mice were housed in standard cages (48 cm × 26 cm). The animals were kept in a room with controlled temperature (22°C) under a 12 h light/12 h dark cycle (lights on at 7 am) and had free access to food and water. The mice (*n* = 40) were randomized into four groups: control (CO), memantine 5 mg (MN5), memantine 10 mg (MN10), and memantine 20 mg (MN20). To avoid social isolation, we maintained two animals per cage (Leasure and Decker, 2009). All behavioral tests were performed between 1:00 pm and 5:00 pm. All experiments were conducted in accordance with official governmental guidelines in compliance with the Federation of Brazilian Societies for Experimental Biology and were approved by the Ethical Committee of the Federal University of Rio Grande do Sul, Brazil.

### Drug Administration

Memantine (Sigma, USA) was dissolved in distilled water at three different concentrations (0.5, 1.0, and 2.0 mg/mL) to standardize the volume used for oral administration and reach the desired dose. For 24 days, the animals received daily administration of either 5, 10, or 20 mg/kg of memantine, or an equivalent volume of distilled water, via oral gavage. Body weight and food intake were monitored. All groups received oral gavage at 1 h after each behavioral task.

### Open Field Test

On the 22nd day, the animals were submitted to an open field task to evaluate spontaneous locomotion and exploratory activity. The apparatus was made of a black-painted box measuring 50 cm × 50 cm and was surrounded by 50 cm high walls. The experiments were conducted in a quiet room under low-intensity light (12 lx). Each mouse (*n* = 10 per group) was placed in the center of the arena, and the distance traveled (total and central zone), time spent in the central zone, and mean speed were measured over a course of 10 min (Muller et al., 2012). The experiment was recorded with a video camera that was positioned above the arena. The analysis was performed using a computer-operated tracking system (Any-maze, Stoelting, Woods Dale, IL, USA).

### Light–Dark Task

On the 23rd day, the light–dark task was performed as previously described (Crawley and Goodwin, 1980) with some modifications to analyze anxiety profiles. The light–dark apparatus consisted of a wood rectangular box with two separated chambers. One chamber had black walls and floor (50 cm × 50 cm × 50 cm) and was not illuminated. The other side had white walls and floor (50 cm × 50 cm × 50 cm) and was illuminated by a 100 W white lamp that was placed overhead. The two compartments were separated by a wall, which had a small opening at floor level. For each experiment, an animal (*n* = 10 per group) was initially placed in the white chamber and then allowed to explore the two-chamber area for a duration of 5 min. The following parameters were recorded by a trained and blinded-to-treatment observer: number of transitions between the two chambers, time spent in the light chamber, and risk assessment behavior. After each experiment, the apparatus was cleaned with 70% alcohol and dried before being used with the next animal.

### Elevated Plus-Maze Task

On the 24th day, the animals were submitted to an elevated plus-maze task to evaluate further signs of anxiety-like behavior. The elevated plus-maze was performed as previously described (Pellow, 1986). The elevated plus-maze apparatus consisted of two open arms (30 cm × 5 cm) and two enclosed arms (30 cm × 5 cm × 10 cm), which were separated by a central platform (5 cm × 5 cm) with the two identical arms of each type being placed opposite to each other. The height of the maze was 70 cm, and the experiments were conducted under dim red light in a quiet room. Each mouse (*n* = 10 per group) was individually placed onto the central platform of the plus-maze, facing one of the open arms, and was observed/recorded for 5 min by a trained and blinded-to-treatment observer. The time spent in the open arms and the total distance traveled were used for further analysis. After each session, the maze was cleaned with 70% ethanol. Data analysis was performed using a computer-operated tracking system (Any-maze, Stoelting, Woods Dale, IL, USA).

### Glutamate Uptake Assay

On the 25th day, the animals (*n* = 6 per group) were sacrificed/dissected and left hippocampal and left frontoparietal cortical brain slices were taken for use in a glutamate uptake assay. The glutamate uptake assay was performed according to Thomazi et al. (2004). Brain hippocampal and frontoparietal cortical slices (0.4 mm) were obtained using a McIlwain tissue chopper and were pre-incubated for 15 min at 37°C in Hank’s balanced salt solution (HBSS), containing 137 mM NaCl, 0.63 mM Na_2_HPO_4_, 4.17 mM NaHCO_3_, 5.36 mM KCl, 0.44 mM KH_2_PO_4_, 1.26 mM CaCl_2_, 0.41 mM MgSO_4_, 0.49 mM MgCl_2_, and 1.11 mM glucose, at pH 7.2. Afterward, 0.66 and 0.33 Ci ml^-1^ L-[^3^H]glutamate were added to a final 100 M concentration of glutamate for incubation with hippocampal and cortical samples, respectively. The incubations were stopped after 5 and 7 min for the hippocampal and cortical samples, respectively, with two ice-cold washes of 1 ml HBSS, which were immediately followed by the addition of 0.5 N NaOH. The samples were kept in this solution overnight. Nonspecific uptake was measured using the same protocol as described above, with differences in temperature (4°C) and medium composition (*N*-methyl-D-glucamine instead of sodium chloride). Na^+^-dependent uptake was considered as the difference between the total uptake and the non-specific uptake. Note that astrocytic transport mediated by GLAST and GLT-1 is responsible for the Na^+^-dependent glutamate uptake (Anderson and Swanson, 2000). Both uptakes were performed in triplicate. Any radioactivity that was incorporated into the slices was measured using a liquid scintillation counter.

### Western Blotting

For western blot analysis, right hippocampal and right frontoparietal cortical homogenates (*n* = 6, per group) were prepared in PIK buffer (1% NP-40, 150 mM NaCl, 20 mM Tris, pH 7.4, 10% glycerol, 1 mM CaCl_2_, 1 mM MgCl_2_, 400 μM sodium vanadate, 0.2 mM PMSF, 1 μg/ml leupeptin, 1 μg/ml aprotinin, and 0.1% phosphatase inhibitor cocktails I and II from Sigma–Aldrich) and centrifuged (Zimmer et al., 2012). Supernatants were collected and total protein was measured using Peterson’s method (Peterson, 1977). Samples containing 20 μg of protein from the hippocampal homogenate were separated by electrophoresis on a polyacrylamide gel and electrotransferred to PVDF membranes. Protein bands within each sample lane were compared to standard molecular weight markers (Precision Plus Protein^TM^ Dual Color Standards, Bio-Rad), which were used to identify the molecular weights of proteins of interest. Non-specific binding sites were blocked using Tween–Tris buffered saline (TTBS, 100 mM Tris–HCl, pH 7.5) with 5% albumin for 2 h. Samples were incubated overnight at 4°C with primary antibodies against GLT-1 (Abcam, 1:1000), GLAST (Abcam, 1:1000), and β-actin (Sigma, 1:5000). Following primary antibody incubation, the membranes were incubated with secondary antibodies (anti-rabbit, GE life sciences, 1:3000; anti-mouse, GE life sciences, 1:5000) for 2 h at room temperature. Films were scanned, and band intensity was analyzed using Image J software (Abramoff et al., 2004).

### Statistical Analysis

Differences between groups were analyzed with analysis of variance (ANOVA) followed by Tukey’s *post hoc* test. Correlations between behavioral assessments and glutamate uptake were analyzed by Pearson’s correlation coefficient. The results are presented as mean values ± SEM. Differences were considered significant at *p* < 0.05.

## Results

### Long-Term NMDAR Antagonism does not Alter Spontaneous Locomotion but Induces Anxiety-Like Behavior

**FIGURE 1:**
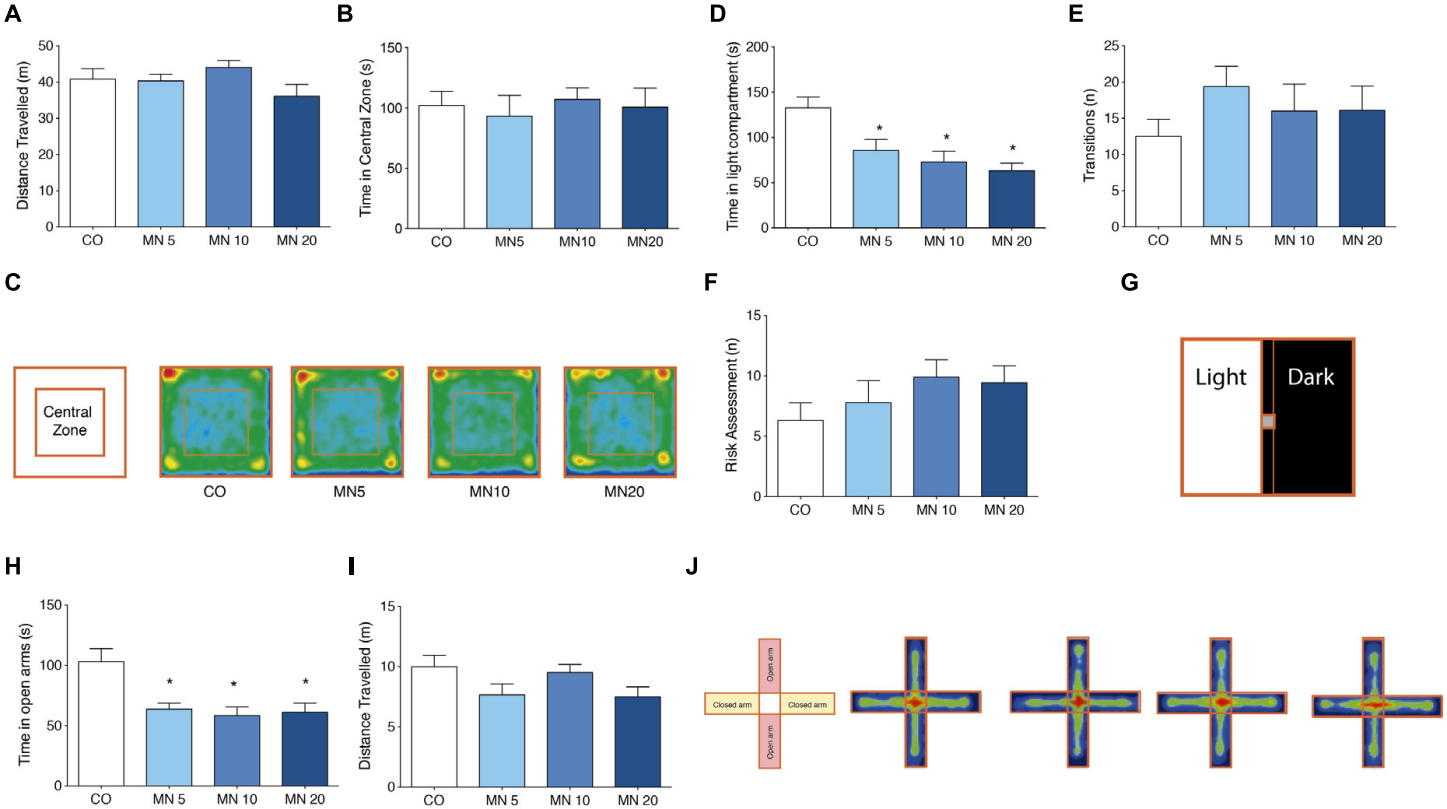
**Long-term NMDAR antagonism does not alter spontaneous locomotor and exploratory behavior, but induces anxiety-like behavior. (A)** Total distance traveled in the open field. **(B)** Time in central zone in the open field. **(C)** Open field apparatus and occupancy plots. **(D)** Time in light compartment in the light–dark box. **(E)** Number of transitions in the light–dark box. **(F)** Risk assessment index in the light–dark box. **(G)** Light–dark box apparatus. **(H)** Time in open arms in the elevated plus-maze. **(I)** Distance traveled in the elevated plus-maze. **(J)** Elevated plus-maze apparatus and occupancy plots. Groups: control (CO), memantine 5 mg (MN 5), memantine 10 mg (MN 10), and memantine 20 mg (MN 20); *n* = 10 per group. Data are presented as mean values ± SEM.

Administration of memantine did not cause significant changes in either distance traveled [**Figure 1A**; *F*_(3,36)_ = 1.642, *p* = 0.1967] or time spent in the central zone [**Figure 1B**; *F*_(3,36)_ = 0.1697, *p* = 0.9162] in the open field. Occupancy plots are used to illustrate the similarities between groups in the open field test (**Figure 1C**).

### Long-Term NMDAR Antagonism Reduced Time Spent in the Light Compartment of the Light–Dark Box

In the light–dark box (**Figure 1G**), all of the doses of memantine that were tested significantly reduced the time spent in the light compartment by the memantine-administered mice compared to the CO group [**Figure 1D**; *F*_(3,36)_ = 7.364, MN5: *p* = 0.03, MN10: *p* = 0.002, MN20: *p* = 0.01]. However, transition numbers (light to dark) were unrelated to memantine administration [**Figure 1E**; *F*_(3,36)_ = 0.8257, *p* = 0.4884]. Additionally, there were no differences among groups in risk assessment index [**Figure 1F**; *F*_(3,36)_ = 1.129, *p* = 0.3519].

### Long-Term NMDAR Antagonism Decreased Time Spent in the Open Arms of the Elevated Plus-Maze

The administration of memantine reduced the time spent by mice in the open arms of the elevated plus-maze (**Figure 1J**) when compared to the CO group [**Figure 1H**; *F*_(3,36)_ = 6.974, MN5: *p* = 0.007, MN10: *p* = 0.002, MN20: *p* = 0.004]; however, there were no changes in total distance traveled [**Figure 1I**; *F*_(3,36)_ = 2.227, *p* = 0.1018].

### Long-Term NMDAR Antagonism Decreased Glutamate Uptake in the Frontoparietal Cortex and Hippocampus without Affecting the Immunocontents of GLAST and GLT-1

**FIGURE 2:**
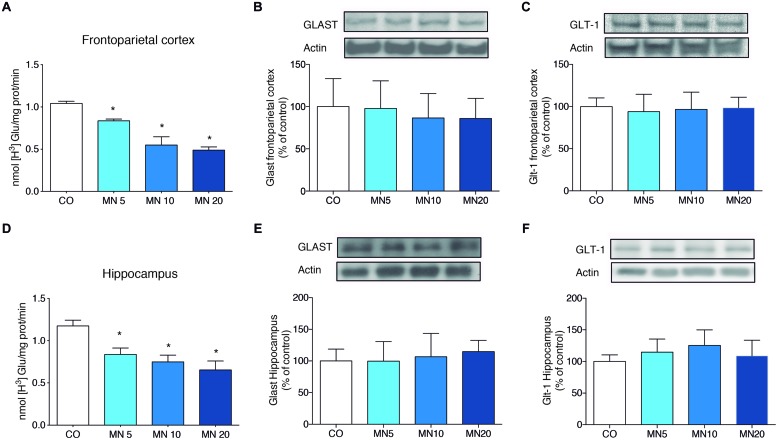
**Long-term NMDAR antagonism decreases glutamate uptake in the frontoparietal cortex and hippocampus, but does not alter astroglial transporter immunocontent. (A)** Glutamate uptake in slices of frontoparietal cortex. **(B)** Immunocontent of GLAST in the frontoparietal cortex. **(C)** Immunocontent of GLT-1 in the frontoparietal cortex. **(D)** Glutamate uptake in slices of hippocampus. **(E)** Immunocontent of GLAST in the frontoparietal cortex. **(F)** Immunocontent of GLT-1T in the frontoparietal cortex. Groups: CO, memantine 5 mg (MN 5), memantine 10 mg (MN 10), and memantine 20 mg (MN 20); *n* = 6 per group. Data are presented as mean values ± SEM. ^∗^*p* < 0.05 among groups.

The administration of memantine significantly decreased glutamate uptake in slices of frontoparietal cortex [**Figure 2A**; *F*_(3,20)_ = 11.458, MN5: *p* = 0.026, MN10: *p* < 0.001, MN20: *p* < 0.001] and hippocampus [**Figure 2D**; *F*_(3,20)_ = 15.008, MN5: *p* = 0.015, MN10: *p* < 0.001, MN20: *p* < 0.001]. However, memantine did not alter the immunocontent of GLAST in either the frontoparietal cortex [**Figure 2B**; *F*_(3,20)_ = 1.300, *p* = 0.3020] or the hippocampus [**Figure 2E**; *F*_(3,20)_ = 0.6174, *p* = 0.6118]. Additionally, no alterations were found in the immunocontent of GLT-1 in either the frontoparietal cortex [**Figure 2C**; *F*_(3,20)_ = 2.225, *p* = 0.1167] or the hippocampus [**Figure 2F**; *F*_(3,20)_ = 0.1520, *p* = 0.9272].

### Correlation Between Anxiety-Like Behavior and Glutamate Uptake

**FIGURE 3:**
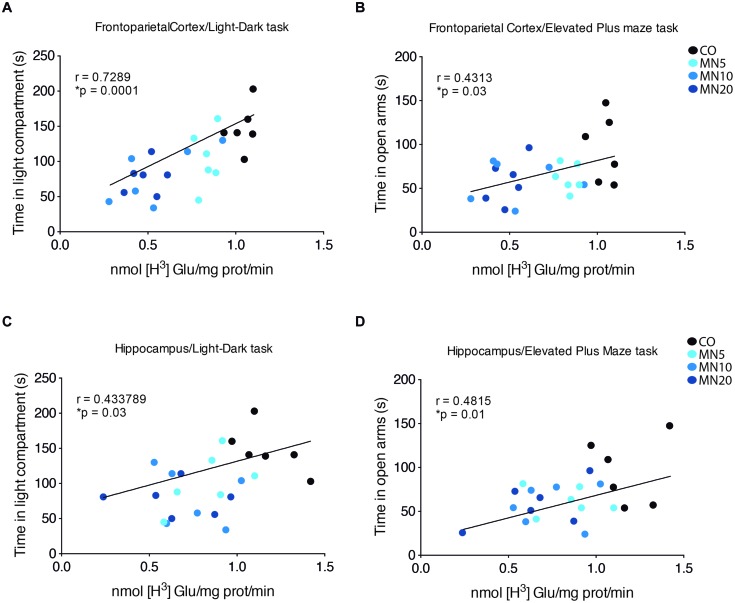
**Anxiety-like behavior correlates with glutamate uptake. (A)** Linear correlation between time spent in light compartment during the light/dark task and glutamate uptake in slices of frontoparietal cortex. **(B)** Linear correlation between time spent in open arms and glutamate uptake in slices of frontoparietal cortex. **(C)** Linear correlation between time spent in light compartment during light/dark task and glutamate uptake in slices of hippocampus. **(D)** Linear correlation between time spent in open arms in elevated plus-maze test and glutamate uptake in slices of hippocampus. Groups: CO, memantine 5 mg (MN 5), memantine 10 mg (MN 10), and memantine 20 mg (MN 20); *n* = 24. Data presented by one animal per point. ^∗^*p* < 0.05.

A positive correlation was found between time spent in the light compartment of the light dark-box and glutamate uptake in the frontoparietal cortex (**Figure 3A**; *p* < 0.0001, *R* = 0.7289) and hippocampus (**Figure 3C**; *p* = 0.03, *R* = 0.4337). Time spent in the open arms of the elevated plus-maze test was also correlated with glutamate uptake in the frontoparietal cortex (**Figure 3B**; *p* = 0.03, *R* = 0.4313) and hippocampus (**Figure 3D**; *p* = 0.01, *R* = 0.4815).

## Discussion

Our results demonstrated that long-term antagonism of NMDAR by memantine induces anxiety-like behavior in healthy CF-1 mice. Additionally, memantine decreased glutamate uptake activity in the frontoparietal cortex and in the hippocampus with this phenomenon correlating with anxiety-like behavior. By contrast, the immunocontents of the astroglial glutamate transporters GLT-1 and GLAST were not affected.

Long-term administration of memantine did not induce significant changes in the spontaneous locomotion and exploratory activity of mice in the open field test. These findings imply that neither the dose nor the regimen of memantine that was used in our work led to non-specific effects such as sedation, which can potentially impair performance in anxiety-like tasks. This finding is in agreement with previous reports that have demonstrated that memantine administration does not alter locomotion or exploratory profiles (Reus et al., 2010). Conversely, we also showed that long-term memantine administration at doses of 5, 10, or 20 mg/kg leads to an anxiogenic phenotype that is manifested by decreased time spent in the light compartment (light–dark box) and reduced time spent in open arms (elevated plus-maze). Interestingly, a previous work showed that the administration of MK801, another non-competitive NMDAR antagonist, to rats induced an anxiety-like phenotype in the elevated plus-maze (Solati, 2011). In contrast to MK801, high doses of memantine (100 mg/kg) increased time spent in open arms, implying an anxiolytic effect. However, doses ranging from 10 to 30 mg/kg decreased time spent in open arms (∼40%), without reaching statistical significance, which suggests a trend representative of an anxiogenic-like effect (Minkeviciene et al., 2008). Additionally, chronic antagonism of NMDAR with piperine18 exacerbated anxiogenic symptoms in C57BL/6 mice (Hanson et al., 2014). Indeed, it would appear that the antagonism of NMDAR does not follow a linear dose-response effect in terms of modulating anxiety-like behavior.

It has also been shown that memantine plays a role in controlling synaptic glutamate release. In fact, Lu et al. (2010) have shown that memantine suppresses glutamate release in cortical synaptosomes. In this study, however, we showed that long-term administration of memantine reduces glutamate uptake without affecting the glutamate transporters expression, GLT-1 and GLAST, in the frontoparietal cortex and hippocampus. Based on these findings, one could argue that memantine-induced reduction of glutamate uptake by astrocytes is a direct adaptive response to the reduced release of glutamate by neurons. This assumption reinforces a theoretical framework in which neurons and astrocytes are capable of sensing each other while regulating tripartite glutamatergic synapses (Wade et al., 2013; Karus et al., 2015). However, further studies using additional methodologies, such as immunostaining and electron microscopy, are necessary to better understand neuron-astrocyte coupling in the context of anxiety-like phenotypes.

Interestingly, a recent work demonstrated that blockade of GLT-1 in the central amygdala was also capable of inducing anxiety-like behavior, which reinforces the association between astrocytic glutamate uptake activity and the development of an anxiety phenotype (John et al., 2015). Remarkably, we were able to show through linear correlation that decreased glutamate uptake activity in the hippocampus and frontoparietal cortex was significantly correlated with an increased anxiety-like response.

## Conclusion

Long-term NMDAR antagonism by memantine induces an anxiety phenotype that is associated with reduced glutamate uptake activity in healthy CF-1 mice, which suggests that interactions between neurons and astrocytes can shape anxiety-related behavior.

## Author Contributions

EZ was responsible for the design, acquisition, analysis, interpretation, drafting, and approval of the final version of the manuscript. VT, EK, MA, KZ, RA, and GH were responsible for acquisition, analysis, interpretation, and approval of the final version of the manuscript. AM, DS, and RV were responsible for interpretation, drafting, critical revision, and approval of the final version of the manuscript. LV was responsible for the design, interpretation, drafting, critical revision, and approval of the final version of the manuscript.

## Conflict of Interest Statement

The authors declare that the research was conducted in the absence of any commercial or financial relationships that could be construed as a potential conflict of interest.
