# Corrigendum: Long-term NMDAR antagonism correlates reduced astrocytic glutamate uptake with anxiety-like phenotype

**DOI:** 10.3389/fncel.2016.00093

**Published:** 2016-04-11

**Authors:** Eduardo R. Zimmer, Vitor R. Torrez, Eduardo Kalinine, Marina C. Augustin, Kamila C. Zenki, Roberto F. Almeida, Gisele Hansel, Alexandre P. Muller, Diogo O. Souza, Rodrigo Machado-Vieira, Luis V. Portela

**Affiliations:** ^1^Department of Biochemistry, Universidade Federal do Rio Grande do SulPorto Alegre, Brazil; ^2^Department of Physiology, Universidade Federal do SergipeSão Cristovão, Brazil; ^3^Laboratory of Exercise, Biochemistry and Physiology, Universidade do Extremo Sul CatarinenseCriciúma, Brazil; ^4^Laboratory of Neuroscience, LIM-27, Institute and Department of Psychiatry, Universidade de São PauloSão Paulo, Brazil; ^5^Center for Interdisciplinary Research on Applied Neurosciences (NAPNA), Universidade de São PauloSão Paulo, Brazil; ^6^Experimental Therapeutics and Pathophysiology Branch, National Institute of Mental HealthBethesda, MD, USA

**Keywords:** anxiety, astrocytes, behavior, glutamate, memantine

In the subsection “Glutamate Uptake Assay,” the greek letter micro (symbol μ) was accidentally deleted twice probably due to software incompatibility. The correct sentence should read: “*Afterwards, 0.66 and 0.33* μ*Ci ml*^−1^*L-[*^3^*H]glutamate were added to a final 100* μ*M concentration of glutamate for incubation with hippocampal and cortical samples, respectively.”* This correction does not affect the scientific relevance of the results.

## Author contributions

EZ was responsible for the design, acquisition, analysis, interpretation, drafting, and approval of the final version of the manuscript. VT, EK, MA, KZ, RA, and GH were responsible for acquisition, analysis, interpretation, and approval of the final version of the manuscript. AM, DS, and RM were responsible for interpretation, drafting, critical revision, and approval of the final version of the manuscript. LP was responsible for the design, interpretation, drafting, critical revision, and approval of the final version of the manuscript.

## Funding

This work was supported by the following Brazilian agencies and grants: National Counsel of Technological and Scientific Development (CNPq), CAPES, FAPERGS, Brazilian Institute of Neuroscience (IBNnet), FINEP, and National Institute of Science and Technology (INCT) – Excitotoxicity and Neuroprotection.

### Conflict of interest statement

The authors declare that the research was conducted in the absence of any commercial or financial relationships that could be construed as a potential conflict of interest.

