# Supplementary material for: Long-term NMDAR antagonism correlates reduced astrocytic glutamate uptake with anxiety-like phenotype
Source: Front Cell Neurosci. 2015 Jun 3;9:219. doi: 10.3389/fncel.2015.00219 (PMC4452887; doi:10.3389/fncel.2015.00219)
Supplement: Supplementary file 1 [file Image_1.PDF]

Supplemental Figure

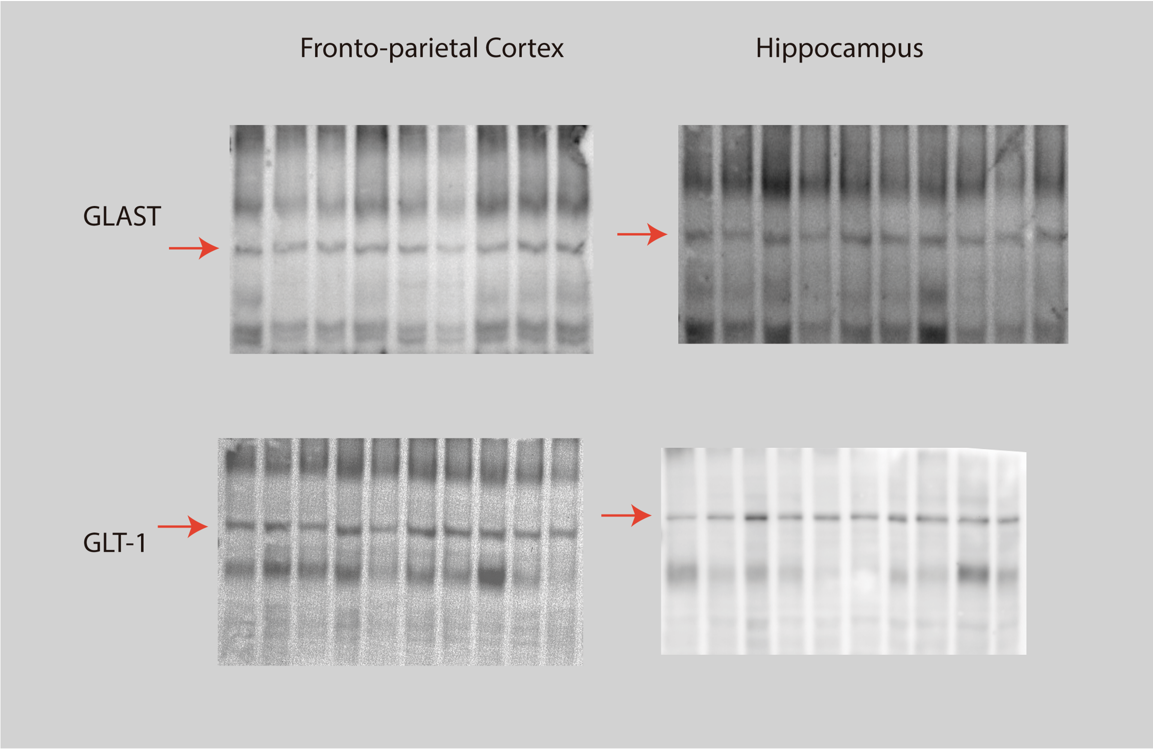

Western blotting membranes

Representative membranes showing the immunocontents of GLAST (a,b) and GLT-1 (c,d) in the frontoparietal cortex and hippocampus, respectively. Antibodies references: GLAST (Anti-EAAT1 antibody, ab416, abcam) and GLT-1 (Anti-EAAT2 antibody, ab41621, abcam). Wells (from left to right): CO, MN5, MN10, MN20, CO, MN5, MN10,MN20, CO, CO.
